# Cognitive dysfunction in hereditary spastic paraplegias and other motor neuron disorders

**DOI:** 10.1590/s1980-5764-2016dn1004004

**Published:** 2016

**Authors:** Ingrid Faber, Lucas Melo T. Branco, Marcondes Cavalvante França Júnior

**Affiliations:** 1 Department of Neurology, School of Medical Sciences, University of Campinas – UNICAMP, Campinas, SP, Brazil.

**Keywords:** hereditary spastic paraplegia, dementia, mental retardation, cognition

## Abstract

Hereditary spastic paraplegia (HSP) is a diverse group of single-gene disorders that share the predominant clinical feature of progressive lower limb spasticity and weakness. More than 70 different genetic subtypes have been described and all modes of inheritance are possible. Intellectual dysfunction in HSP is frequent in recessive forms but rare in dominant families. It may manifest by either mental retardation and/or cognitive decline. The latter may be subtle, restricted to executive dysfunction or may evolve to severe dementia. The cognitive profile is thought to depend largely on the genetic subtype of HSP, although wide phenotypic variability within the same genetic subtype and also within the same family can be found.

## Abstract (pt)

As paraplegias espásticas hereditárias (PEH) constituem um grupo heterogêneo de doenças monogenicamente determinadas que compartilham o aspecto clínico predominante de espasticidade e fraqueza progressivos. Mais de 70 subtipos genéticos já foram identificados, sendo que todos os modos de herança são possíveis. Disfunção intelectual é frequente nas formas de herança autossômica recessiva, enquanto nos subtipos dominantes sua ocorrência é considerada rara. Tais transtornos podem se manifestar como retardo mental e/ou declínio cognitivo progressivo. O último pode ser leve, restrito a disfunção executiva, ou evoluir para demência incapacitante. Acredita-se que o perfil cognitivo dos pacientes dependa grandemente do subtipo genético, contudo, grande variabilidade fenotípica pode ser verificada dentro de um mesmo subtipo e mesmo dentro da mesma família com paraparesia espástica hereditária.

## INTRODUCTION

Hereditary spastic paraplegia (HSP) is a diverse group of single-gene disorders that share the predominant clinical feature of progressive lower limb spasticity and weakness. Signs and symptoms are caused by distal axonopathy of the corticospinal tract, a process that can be triggered by more than 70 different genetic subtypes described to date.^1,2^ These may segregate as an autosomal dominant, autosomal recessive, X-linked or mitochondrial trait. Pure forms of the disease are usually autosomal dominant (AD-HSP). Autosomal recessive HSPs (AR-HSPs) on the other hand, predominantly have a complex clinical picture, comprising peripheral neuropathy, movement disorders, cognitive dysfunction and a variety of other features.^3,4^

Population-based studies indicate a prevalence rate of HSPs of approximately 4.1-7.4/100,000.^5,6^ AD-HSPs are known to be more frequent than AR-HSPs and account for up to 80% of cases in Europe and North-America. In Brazil, there is evidence suggesting that recessively inherited HSP is at least as common as AD-HSPs (unpublished data), possibly explained by the high frequency of consanguineous marriages.

Intellectual dysfunction in HSP is frequent in recessive forms but is considered rare in dominant families. It may manifest by either mental retardation and/or cognitive decline. The latter may be subtle, restricted to executive dysfunction or evolve to severe dementia. The cognitive profile is thought to depend largely on the genetic subtype of HSP. Nonetheless, no large studies have been conducted to investigate the frequency of the disorder or characterize predominantly affected cognitive domains. The vast majority of papers on this topic are limited to single individuals or kindred.

## COGNITIVE DYSFUNCTION IN AD-HSPS

HSP-SPG4 is the most frequent genetic subtype, responsible for 35-50% of dominant forms.^7^ It usually presents with a pure clinical picture but there are rare families described with a variety of cognitive problems. The largest study regarding this matter was performed by Tallaksen et al. involving 10 HSP-SPG4 families. The authors demonstrated subclinical executive dysfunction in affected patients when compared to other family members that did not carry the mutation.^8^ Mood and behavioral issues are frequently neglected in HSP and other primarily motor neurodegenerative conditions. A study investigating non-motor features in a cohort of 30 HSP-SPG4 revealed higher rates of depressive symptoms in patients when compared to controls, with 36.6% diagnosed with depression. Pain and fatigue were also frequent manifestations of the disease.^9^

McMonagle et al. found progressive cognitive decline in 3 families with HSP-SPG4, where 7 of the 11 patients followed were diagnosed as having dementia.^10^ Murphy and colleagues prospectively investigated a large family with HSP-SPG4 in which progressive cognitive deterioration was observed. Characterized initially by dysexecutive syndrome, this later affected memory, perceptual judgment and ultimately led to disorientation and poor interaction. Some members of this kindred also harbored a mutation in the *SPG6* gene. This mutation was claimed by the authors not to be associated with dementia, since one family-member harboring only an *SPG6* mutation was cognitively healthy. One patient was submitted to postmortem pathological examination. The spinal cord was atrophic but cerebral structures were normal on macroscopy. The most significant microscopic changes were widespread white and grey-matter ubiquitin-positive grains.^11^ White et al., on the other hand, demonstrated a wide assortment of pathological changes affecting an HSP-SPG4 individual. These included neuronal depletion and tau-immunoreactive neurofibrillary tangles in the hippocampus as well as cortical balloon cells immunoreactive for tau. Lewy bodies were seen in the substantia nigra.^12^ The proband manifested slowly progressive spastic gait since age 18. Around his seventies, he developed dementia (Mini-Mental State Examination Score 13/30) comprising memory impairment, naming difficulties, ideomotor apraxia and perseveration. Two deceased members of his family had a history of memory impairment and another had learning difficulties. Webb et al. investigated a large family with AD-HSP linked to the *SPG4* locus. Of 11 affected members, the 4 eldest exhibited global cognitive decline, but this finding was also observed in 1 of the 11 unaffected family members, indicating that this may have been a coincidental finding.^13^

No consistent explanation as to why the vast majority of HSP-SPG4 families do not have obvious intellectual impairment has yet been put forward. One possibility is that the HSP-SPG4 patients described with late-life dementia could in fact also have a coincidental, more common disease, such as Alzheimer's or Vascular Dementia. Another hypothesis is that *SPG4* mutations might act as a risk factor that can, in association with other elements, trigger multifactorial degenerative dementias.

Mental Retardation in HSP-SPG4 has been reported to be associated with posterior fossa congenital abnormalities.^14^ Ribai et al. described 13 patients from three HSP-SPG4 families with associated mental handicap and no structural brain anomalies. Deficits ranged from social dependence to severe mental retardation with absence of language and dependence for activities of daily living. Mutations described in these families are also found in other kindreds with pure HSP, indicating that the mutation type is probably not the determinant factor causing psychomotor delay. Some intragenic polymorphisms are associated with pure, but early onset HSP-SPG4. In keeping with this, intellectual deficiency could be imputed to a similar molecular mechanism. Favoring this hypothesis, *SPG4* segregated both with spastic gait and mental deficiency in Ribai's cohort.^15^

In summary, cognitive complaints are probably not a frequent finding in HSP-SPG4 families, although comprehensive neuropsychological testing can reveal subtle executive dysfunction in some cases. On the other hand, subcortical dementia or mental retardation, although rare, should not detract the clinician from this diagnosis.

HSP-SPG10 is the dominant form most commonly associated with complicated features, especially peripheral neuropathy, in up to two third of cases. Other relatively common findings, both affecting approximately 12% of patients (25% of families), include mental retardation and parkinsonism.^16^

## COGNITIVE DYSFUNCTION IN AR-HSPS

Intellectual disability is a frequent finding in AR-HSP. There is a wide phenotypic variability within the same genetic subtype and even within the same family. The two most frequent AR-HSPs are SPG7 and SPG11.^7,17^ The former typically manifests in mid-adult life with either a spastic or ataxic gait. Key findings that suggest this diagnosis are ptosis, progressive external ophthalmoplegia and optic atrophy. Intellectual disability is not reported in HSP-SPG7, although impaired emotional communication and executive dysfunction have been recently described in one patient.^18^

HSP-SPG11 is characterized by spastic paraparesis starting around the second decade of life. Many families report a history of low academic performance heralding gait abnormalities. Key diagnostic findings include thinning of the corpus callosum (TCC) and frontal white matter lesions with a typical radiological appearance called the "Ears of Lynx sign".^7^ The disease progresses relatively rapidly in comparison with other HSPs, with most patients becoming wheelchair bound after 1 or 2 decades of the disease. The few cases described in the fifth decade of life are tetraplegic and demented, with little or no voluntary speech.^19^ The phenotype is invariably complicated, including: ataxia, parkinsonism, dystonia, peripheral neuropathy (that can mimic amyotrophic lateral sclerosis), epilepsy, low visual acuity and others. ^19^ Neuroimaging studies provide in vivo evidence that neuronal damage is not restricted to corticospinal tracts in HSP-SPG11, with significant volumetric reduction affecting periventricular white matter and deep gray matter.^20^

Cognitive disability is described in 80-100% of HSP-SPG11 cases, but its characteristics have yet to be elucidated.^17,21^ It is unclear whether these patients present with congenital mental impairment, progressive cognitive decline or a mixture of both. Reduced verbal fluency is a striking finding that sometimes leads to mutism.^19^ Siri et al. performed a comprehensive neuropsychological assessment in two HSP-SPG11 patients. The evaluation results were consistent with executive dysfunction, encompassing memory consolidation, attention, visual perception and organization problems. In our study group, unpublished data support the hypothesis of a progressive disturbance resembling a frontotemporal dementia syndrome. Behavioral issues such as aggressiveness and psychosis have been described.^19^ An infantilized and silly nature with frequent smiling seems to be the commonest pattern, resembling a pseudobulbar affect but only with the jocular component (author's observations).

TCC can be encountered in other, rarer AR-HSPs, including: *SPG15, SPG21, SPG35, SPG46, SPG47, SPG48, SPG54, SPG55, SPG56, SPG64, SPG73* and others. TCC is not a constant in all these genetic subtypes, but when present, is usually accompanied by mental incapacity. The relationship between this structural abnormality and mental handicap, however, is not well defined. Intellectual deficiency is a variable finding that can be present even in the absence of MRI abnormalities. clearly defined dementia seems to be less frequent in non-SPG11 HSPs.^17,19^

## COGNITIVE DYSFUNCTION IN X-LINKED HSPS

X-linked HSPS are rarer still, and the few clinical reports highlight significant mental retardation associated with early onset or congenital spasticity. HSP-SPG1 and HSP-SPG2 are the commonest subtypes. HSP-SPG1 is usually associated with hydrocephalus and classically presents as MASA syndrome, an acronym for: Mental retardation, Aphasia, Shuffling gate and Adducted thumbs. Agenesis of the corpus callosum is another classical finding.^22^ HSP-SPG2 is allelic to Pelizaeus-Merzbacher, a hypomyelinating congenital disorder/leukodystrophy. In addition to spastic paraplegia, severely affected patients may display mental retardation, optic atrophy, ataxia and white matter lesions.^23^ As a sign of the extreme phenotypic heterogeneity that characterizes this group of diseases, pure HSP-SPG2 has also been detected.

## COGNITIVE DYSFUNCTION IN OTHER MOTOR-NEURON DISORDERS

Other motor-neuron diseases share phenotypic and genetic similarities with HSP, notably the juvenile-onset forms of amyotrophic lateral sclerosis (JALS), a very rare condition with onset before age 25 years.^24^ These usually have a slow rate of progression and variable symptoms, according to the genotypic profile, and are frequently related to familial inheritance but also associated with de novo mutations. There are six familial ALS types allegedly linked with JALS, which correspond to mutations in the following genes: *ALS2* (ALS type 2), *SETX* (ALS type 4), *SPG11* (ALS type 5), *FUS* (ALS type 6), *UBQLN2* (ALS type 15) and *SIGMAR1* (ALS type 16).^25,26^

Cognitive and behavioral alterations are seldom reported, related only to specific JALS types. *FUS* and *UBQLN2* mutations have been linked with adult-onset ALS with frontotemporal dementia,^27,28^ but only the first type is allegedly associated with mental retardation in JALS reports.^29,30^ A JALS/dementia cluster has also been described in Dutch patients, corresponding to an unidentified autosomal recessive form (OMIM %205200).^31,32^ Regarding behavioral changes, JALS type 2 patients had features of pseudobulbar affect in a Tunisian family.^33^ To date, there have been no reports of cognitive or behavioral changes in the remaining JALS types. However, it is important to highlight that there is a lack of large cohort-based studies investigating the JALS types.

## CONCLUSION

In summary, cognitive dysfunction is highly variable in HSP. Autosomal dominant families typically do not display clear impairment but recent evidence suggests the possibility of mild executive dysfunction in HSP-SPG4. The possibility of late-life dementia should be the subject of further investigation. Intellectual disability is a frequent finding in Autosomal Recessive and X-linked HSPs. In HSP-SPG11, the disorder is probably progressive, with significant impairment of executive abilities. HSP is a highly heterogeneous group of disorders and longitudinal studies are warranted to further characterize cognitive and behavioral dysfunctions as either developmental or neurodegenerative. Such findings could evolve to become a valuable parameter, together with neuroimaging and other biomarkers, to help better understand the pathophysiology of the disease.
